# Intratumoral C3ar/C5ar1 Antagonists Imbedded in an In Situ Forming Implant Can Robustly Suppress Solid Tumors

**DOI:** 10.3390/cells15110971

**Published:** 2026-05-22

**Authors:** Young A Choi, Ryan Konrad, Elliot S. Pohlmann, Eric Abenojar, Agata Exner, M. Edward Medof

**Affiliations:** 1 Institute of Pathology, Case Western Reserve University, Cleveland, OH 44106, USA; young.choi@advocatehealth.org (Y.A.C.); ryan.konrad@case.edu (R.K.); elliot.pohlmann@case.edu (E.S.P.); 2 Virginia Tech Carilion School of Medicine and Research Institute, Roanoke, VA 24016, USA; eric.abenojar@case.edu (E.A.); agata.exner@case.edu (A.E.)

**Keywords:** C5a, C5ar1 signaling, cancer viability

## Abstract

Solid tumors typically expand in a “cold” immunosuppressive tumor microenvironment (TME) and resist killing by CAR T cells or conventional therapy. Herein, we show that intratumoral injection of C3a and C5a receptor 1 (C3ar/C5ar1) pharmaceutical antagonists in an in situ forming implant (ISFI) can robustly suppress such tumors. Antagonizing autocrine C3ar/C5ar1 signaling in eight human and murine cancers of diverse lineages was universally anti-mitotic and pro-apoptotic in vitro, and growth-repressive in vivo. In contrast to i.p. administration of C3ar/C5ar1 antagonists to tumor-bearing mice, injecting the antagonists intratumorally in slow release poly (lactic-co-glycolic acid) (PLGA) polymer caused near-complete tumor elimination. The focused blockade of C3ar/C5ar1 GPCR signaling in an intratumoral ISFI opposed solid cancers by jointly repressing cancer cell viability/growth, tumor-associated angiogenesis, and myeloid-derived suppressor cell (MDSC) recruitment. Thus, the sustained blockade of C3ar/C5ar1 signaling in an intratumoral ISFI uninterruptedly disrupts three processes essential for solid cancer growth while avoiding adverse effects on other cell types. Our findings may apply to multiple cancer types in which discrete tumor masses can be targeted.

## 1. Introduction

Accumulating evidence has implicated the complement activation fragments C3a and C5a in modulating the growth of several cancer types [1,2,3,4]. C3a/C5a, traditionally presumed to derive from liver-produced complement components in plasma, are now regarded in some cases to also come from C3/C5 proteins produced by tumors or by immune and/or other cells in the tumor microenvironment (TME). Some data point to the C3a/C5a functioning endogenously in the cells and in plasma [5,6,7]. Noncanonical effects on the tumor and/or on cells in the TME have been described [1,2,3,4]. The tumor modulatory functions of the C3a/C5a, however, remain incompletely characterized. Both tumor-suppressing and tumor-promoting effects of their blockade have been reported [8], limiting clinical translation.

While cytotoxic T effector cell (Teff) responses against tumors are able to evoke tumor inhibition or elimination in many cancers, many data have shown that myeloid-derived suppressor cells (MDSCs) in the TME characteristically subvert these Teff responses. Supplementing anti-cancer regimens with checkpoint inhibitors that can interdict the immunosuppressive activities of the inhibitory cells has improved therapeutic outcomes, but some cancers still accumulate MDSCs and resist elimination. TME-associated C3a/C5a fragments have been implicated in contributing to this resistance.

Relevant to the currently known cellular functions of C3a/C5a, we and others [5,7,9,10,11] have previously showed that they normally play a role in regulating T-cell responses. While our studies in multiple models of infection, autoimmunity, and transplantation showed that C3ar/C5ar1 signaling [7,12,13] promotes Teff responses, analyses of processes tied to transplant survival induced by costimulatory blockades paradoxically showed that autocrine C3ar/C5ar1 signaling in MDSCs is essential for the recruitment of these immunosuppressive cells [14].

Based on the emerging data on complements’ effects in cancer [15] and the above linkage of autocrine C3ar/C5ar1 signaling with MDSC recruitment, we investigated whether the potentiation or suppression of GPCR signaling analogously regulates cancer cell growth. In vitro and in vivo studies of four human and four murine cancer lineages showed that autocrine C3ar/C5ar1 signaling uniformly operates in multiple cancer types, is pro-mitotic/anti-apoptotic, and that disabling it markedly represses cancer cell viability as well as growth. These findings prompted the question of how modulating C3ar/C5ar1 signaling in vivo would impact tumor progression. The investigation of this issue yielded the critical insight that focusing on C3ar/C5ar1 blockade in the tumor and the TME by the intratumoral injection of C3ar/C5ar1 pharmaceutical antagonists in an in situ forming implant (ISFI) jointly interfered with multiple processes that enable tumor progression, and thereby significantly augmented tumor elimination, a protocol that could be broadly applicable to multiple solid cancer cell types.

## 2. Materials and Methods

### 2.1. Reagents and Antibodies

Murine C5a was purchased from Cell Sciences (Canton, ME, USA). The C3ar1 antagonist (C3ar-A) and C5ar1 antagonist (C5ar1-A) are from Calbiochem (EMD Millipore, Billerica, MA, USA), catalog numbers C3ar1-A 559410-10mg and C5ar1-A-234415-5mg. Anti-C3a and anti-C5a mAbs were purchased from BD Biosciences (San Diego, CA, USA). Anti-C3ar and anti-C5ar1 were purchased from Santa Cruz Biotech (Santa Cruz, CA, USA).

### 2.2. Cell Lines

The B16 murine melanoma, TC-1 murine lung carcinoma, MCA205 murine fibrosarcoma, and Eo771 murine breast cancer were obtained from the ATCC and either used immediately for experimentation or aliquoted to freezer plugs upon receipt. Human HL60 lung cancer, A375 melanoma, G98T brain cancer, and MDA-MB 468 breast cancer were similarly obtained and handled. Freezer plugs were thawed immediately prior to experimentation. HUVECs were obtained from Lonza.

### 2.3. Cell Growth and Viability Assays

The murine B16 melanoma, TC-1 lung carcinoma, and MCA205 fibrosarcoma cell lines were grown in RPMI 1640 supplemented with 10% heat-inactivated fetal bovine serum (Hy clone), penicillin/streptomycin (ThermoFisher, Carlsbad, CA, USA), and L-glutamine (ThermoFisher Carlsbad, CA, USA). The murine Eo771 breast cancer line was grown in Dulbecco’s Modified Eagle Medium (DMEM) supplemented with 10% heat-inactivated fetal bovine serum, penicillin/streptomycin, and L-glutamine. HUVEC were grown in EC media (Lonza, Basel, Switzerland). For quantifying growth, cells were plated in 24-well plates, starved overnight in 0.5% FBS media, and stimulated with or without C5a (100 ng/mL) or C3ar-A/C5ar1-A (10 ng/mL each) and counted over 72 h using trypan blue to detach adherent cells and exclude dead cells. The same conditions were used for studying the human cancer lines.

For viability studies, cells were allowed to adhere overnight in Falcon 24-well plates in their respective medium containing 10% heat-inactivated FBS, L-glutamine and pen/strep, and then starved overnight in their respective serum-free medium containing 0.5% heat-inactivated FBS, L-glutamine and pen/strep. Following a Dulbecco’s PBS washing step, the conditioned medium used for growth assays was prepared by adding C5a (diluted to the desired concentration) or C5ar1-A/C5ar1-A (10 ng/mL) to the starvation medium in a 50 mL falcon tube and then adding aliquots to starved cells. Cells were then incubated for indicated times and cell numbers quantified using a Bright-Line^™^ hemocytometer (Sigma, St. Louis, MO, USA) distinguishing live and dead cells by trypan blue exclusion (ThermoFisher, Carlsbad, CA, USA). For Annexin V assays, cells (plated in 24-well plates at 5 × 10^5^ cells/well) were treated with C3ar-A/C5ar1-A (10 ng/mL each) or anti-C3a/anti-C5a mAbs (10 μg/mL each). The treated cells were stained with either propidium iodide (PI), Annexin-V-FITC, or both, after which they were incubated for 10 min at 20 °C in the dark. Flow cytometric assays were done on an LSR II flow cytometer (BD Pharmingen, San Diego CA, USA).

### 2.4. qPCR, ELISAs, and Immunoblots

RNA was prepared from TRIzol (Invitrogen, Carlsbad, CA, USA) extracts of cells. cDNAs were synthesized by incubating 1 μg of total cellular RNA in 20 μL of M-MLV Reverse Transcription mixture (Invitrogen, Carlsbad, CA, USA) according to the manufacturer’s instructions. A total of 2 μL of cDNA was mixed with 1 μL of primer and 0.4 μL of Choice *Taq* Blue DNA Polymerase (Denville Scientific Inc, South Plainfield, NJ, USA) and amplified in a MJ Research Inc. (Waltham, MA, USA) PTC-0200 DNA Engine using a 96v Alpha Unit heating block and an annealing temperature of 60 °C. Amplification was assayed by 4% Agarose Gel Electrophoresis. For qPCR, 10 μL of diluted cDNA was mixed with 1 μL of primer and 10 μL SYBR green master mix (Applied Biosystems, Foster City, CA, USA) and amplification assayed on an ABI Prism 7000 Cycler. In all assays, fold increases are relative to the basal level and standardized to actin. Primer sequences for complement genes used in this study are qm B-Actin forward: 5′—CTA AGG CCA ACC GTG AAA AQG- 3′, qm-B-Actin reverse: 5′—ACC AGA GGC ATA CAG GGA CA—3′; qm-C3 forward: 5′—ACC TTA CCT CGG CAA GTT TCT—3′, qm-C3 reverse: 5′-TTG TAG AGC TGC TGG TCA GG-3′; qm-C5 forward: 5′—GGA TTC AAG CGC ATA ATA GCA—3′, qm-C5 reverse: 5′—ACC CGG ATG TTG ACT CCT C—3′; qm-fD forward: 5′—CTG GGA GCG GCT GTA TGT—3′, qm-fD reverse: 5′—CAC GGA AGC CAT GTA GGG—3′; qm-fB forward: 5′—CTC GAA CCT GCA GAT CCA C—3′, qm-fB reverse: 5′—TCA AAG TCC TGC GGT CGT—3′. Primer sequences for pro- and anti-apoptotic factors, as mBcl-2 forward: 5′—GAC GTC TCC TCT CAG GCC CC-3′, reverse: 5′—TCA CGA CGG TAG CGA CGA GA—3′, mouse Bcl-xL [16] forward: 5′-GGTCGCATCGTGGCCTTT–3′ and reverse: 5′-TCCGACTCACCAATACCTGCAT-3′, mouse Bim [17] forward: 5′-CGACAGTCTCAGGAGGAACC-3′, reverse: 5′-CCTTCTCCATACCAGACGGA-3′ and mouse Bax forward: 5′—CTG ACC TTG GAG CAG CCG CC—3′ and reverse: 5′—GTC CAC GTC AGC AAT CAT CC—3′.

Serum-free culture supernatants were isolated from human and murine cancer cell cultures and concentrated using Centricon centrifugal filters (Burlington, WI, USA, Millipore). The concentrated murine culture supernatants were assayed for endogenous C5a in the ELISA protocol described previously [7]. The concentrated human culture supernatants were assessed using a human C5a ELISA kit (R&D Systems, Minneapolis, MN, USA) per the manufacturer’s protocol.

For immunoblots, cells were harvested at indicated time points post-treatment. Equal concentrations of cell lysates were loaded and separated by SDS PAGE using 10% Tris-HCl gels and blotted onto PVDF membranes. Blots were probed with an anti-mouse p-AKT antibody (Cell Signaling Technology) and a HRP-conjugated secondary antibody, and visualized with ECL (Amersham Biosciences, Piscataway, NJ, USA). Blots were re-probed with anti-mouse total AKT antibody (Cell signaling technology, Danvers, MA, USA) as a loading control.

### 2.5. Animals and In Vivo Experiments

C57BL/6 WT, SCID, *C3^-/-^*, and *CD11b^-/-^* mice were purchased from Jackson Labs (Bar Harbor, ME, USA). Mice were inoculated subcutaneously with 2 × 10^4^ cells/100 μL HBSS (Hank’s balanced salt solution) i.p. C3ar-A/C5ar1-A (1 mg/kg each in HBSS) were injected i.p. every second day, and anti-C3a/anti-C5a mAbs (1 mg/kg each in HBSS) were given i.p. weekly. Weight, movement, and eating were monitored every other day as measures of rodent health, and any animal in distress was euthanized. All studies were approved by the Institutional Animal Care and Use Committee (IACUC).

### 2.6. Statistics

Statistical significance for all growth curves was determined by Student’s *t* test and determined for survival by Kaplan–Mayer analysis. All experimentation was performed in triplicate at least 2 or 3 times. Data are presented as standard error mean (SEM) ± standard deviation.

## 3. Results

### 3.1. Cancer Cells Generate C3a/C5a Which Signal Through Their C3ar/C5ar1 to Drive Their Growth

**Figure 1:**
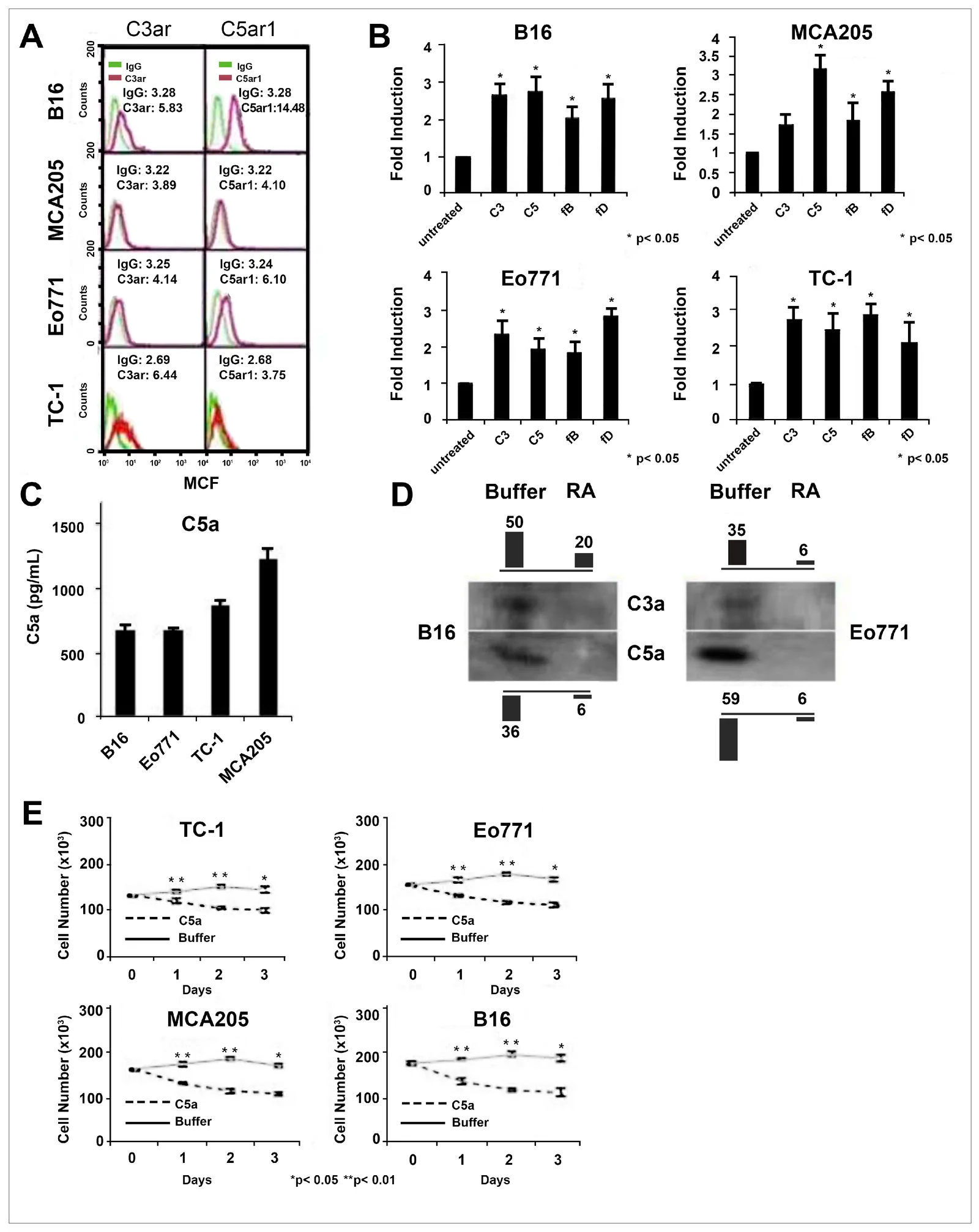
Autocrine C3ar/C5ar1 signaling operation in murine cancer cell lines: (**A**) flow cytometric assays of B16 melanoma, MCA205 fibrosarcoma, Eo771 breast carcinoma, and TC-1 lung carcinoma lines stained for C3ar and C5ar1 [IgG control (gray line) and C3ar and C5ar1 mAbs (black lines)]. MFI = mean fluorescent intensity. Each analysis is representative of at least two replicate assays. (**B**) 1 × 10^5^ cells of each line were incubated at 37 °C with either 100 ng/mL of C5a or media with heat-inactivated FBS alone for 1 h, after which their RNA was assayed for complement transcripts by qPCR. (**C**) Each cancer line was cultured for 24 h in 0.5%-heat-inactivated serum medium and C5a in the culture supernatant was assayed by ELISA. (**D**) B16 and MCA205 lines (1 × 10^5^ cells) were incubated in the absence or presence of C3ar-A/C5ar1-A (RA) 10 ng/mL, each for 6 h, after which cell extracts were immunoblotted for C3a and C5a. Results are given as mean ± SD. (**E**) Each line was incubated in the absence (solid line) or presence (dotted line) of 100 nM C5a in 0.5% heat-inactivated FBS, and cell numbers were quantitated over 3 days. * *p* < 0.05, ** *p* < 0.01 vs. buffer controls.

To determine whether autocrine C3ar/C5ar1 signaling operates endogenously in cancer cells, similarly to in immune cells, and establish whether the process applies broadly to different cancer types, we examined murine cancer lines of four different lineages for expression of C3ar and C5ar1, production of C3/C5 mRNA transcripts, and generation of the C3ar/C5ar1-activating ligands C3a/C5a. All four lines expressed both receptors (Figure 1A), all tonically generated an alternative pathway (AP) (C3/fB/fD) and C5 transcripts (Figure 1B and Figure S1A), and all produced C3a (not shown) and C5a activation fragments (Figure 1C). Consistent with auto-inductive C3ar/C5ar1 signaling operating in the cancer cells, adding C5a elicited the generation of more C5a in a C3ar/C5ar1-dependent manner (Figure 1D). In accordance with heightened autocrine C3ar/C5ar1 signaling playing a role in cancer cell growth, adding C5a accelerated the ex vivo growth of each cancer type (Figure 1E).

### 3.2. Autocrine C3ar/C5ar1 Signaling Provides Requisite Viability Signals to Cancers

**Figure 2:**
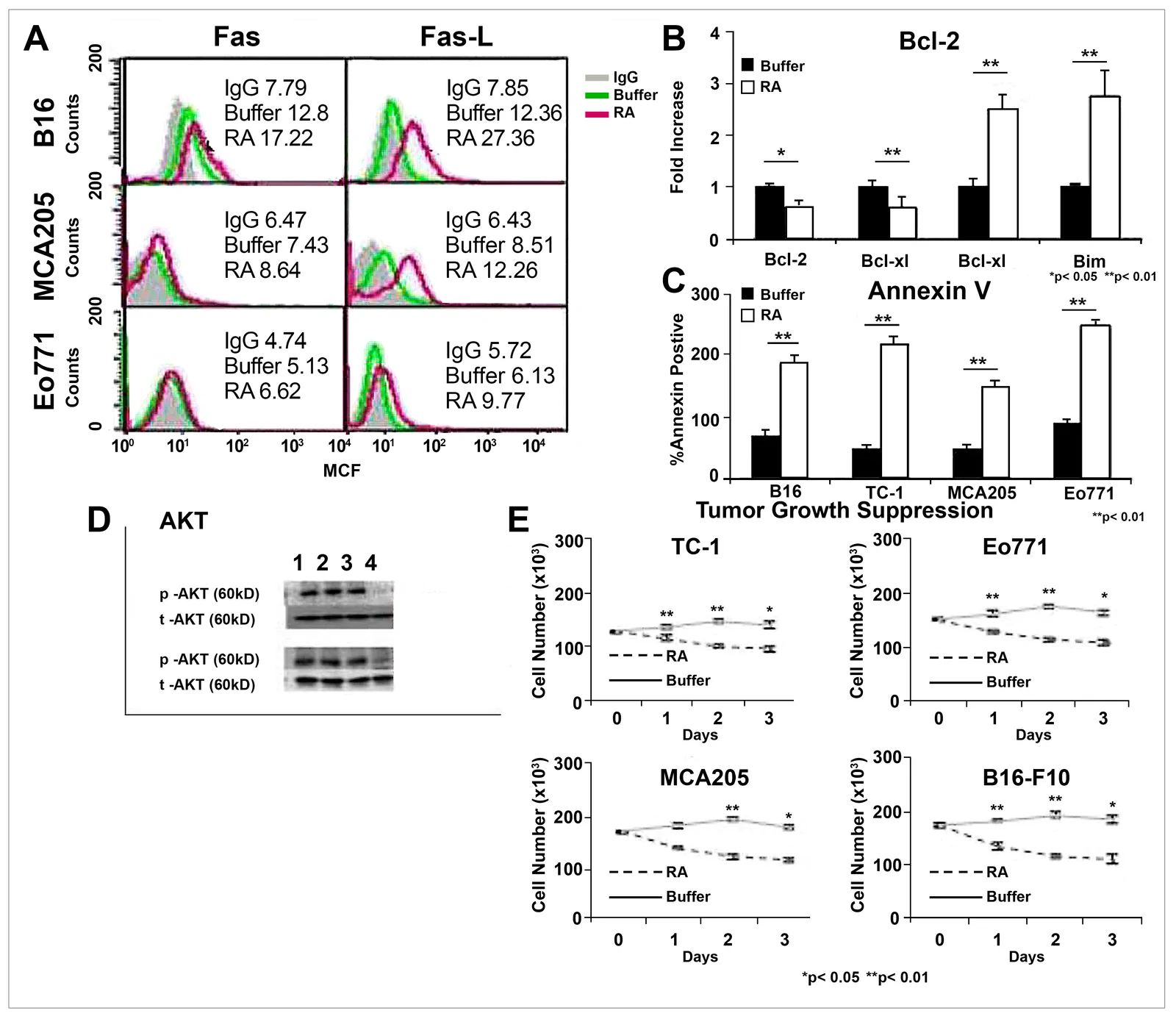
Disabling C3ar/C5ar1 signaling in murine cancers triggers apoptosis. (**A**) Each cancer cell line (1 × 10^5^ cells) was incubated for 6 h with or without C3ar-A/C5ar1-A (RA) (10 ng/mL each), after which Fas and FasL expression were assessed by flow cytometry. Solid light gray area = IgG staining control, green line = untreated (buffer) methanol-treated cells, black line = C3ar-A/C5ar1-A (RA)-treated cells. Experiments are representative of two or more replicate studies. (**B**) Each cancer line was treated as in (**A**) and its RNA was assessed for anti-apoptotic factors, Bcl-2 and Bcl-xL, and pro-apoptotic factors, Bax and Bim, by qPCR. (**C**) Each cancer line was treated as in (**A**) and assessed by flow for Annexin-V-FITC staining using an Annexin V-PI staining kit (BD Biosciences 556419. * *p* < 0.05, ** *p* < 0.01 vs buffer controls. (**D**) B16 and MCA205 cells (1 × 10^5^ cells) were incubated with C3ar-A/C5ar1-A (RA) (10 ng/mL each) for 6 h, after which cell extracts were immunoblotted with anti-phospho-Ser^473^ AKT or total AKT mAb. M: marker, 1: buffer, 2: IgG, 3: MeOH, and 4: C3ar-A/C5ar1-A. (**E**) Each murine cancer line was seeded overnight at 5 × 10^5^ cells/mL, starved for 24 h, incubated with the buffer alone (MeOH vehicle control) or C3ar-A/C5ar1-A (10 ng/mL in MeOH) and growth quantified over 72 h * *p* < 0.05, ** *p* < 0.01 vs. buffer controls.

We next examined whether C3ar/C5ar1 signaling not only promotes growth, but also importantly provides requisite viability signals to the cancer cells. To test this, we utilized a combined C3ar/C5ar1 pharmaceutical antagonist (C3ar-A/C5ar1-A) blockade in view of our previous findings in immune cells [7,12] that C3ar and C5ar1 ligations overlap in their downstream signaling, and that a blockade of each receptor individually has a partial effect. In all four murine cancer lines, including C3ar-A/C5ar1-A in the cultures, upregulated surface expression levels of Fas and FasL were observed in their cultures (Figure 2A and Figure S1A), as previously found in immune cells [7,11]. It concomitantly reduced Bcl-2/Bcl-xl and reciprocally upregulated Bax/Bim mRNA levels (Figure 2B). Annexin-V uptake verified that the PCD-relevant alterations promoted apoptosis (Figure 2C). This result parallels our previous finding in immune cells and cultured cancer cell lines that tonic C3ar/C5ar1 signaling participates in sustaining their survival [7,11,18]. In line with this effect, adding C3ar-A/C5ar1-A to the cancers suppressed AKT phosphorylation (Figure 2D) and inhibited their growth (Figure 2E). The antagonists not only block PI3k-AKT-mTOR signaling, but also STAT3 and ERK [7,13,18]. Taken together, the data argued that autocrine C3ar/C5ar1 signaling constitutes a common viability [7,11,18], in addition to a mitotic signaling loop shared by many cancer lineages, and that its disruption can oppose their growth.

### 3.3. Autocrine C3ar/C5ar1 Signaling Sustains the Viability of Human Cancer Cells

**Figure 3:**
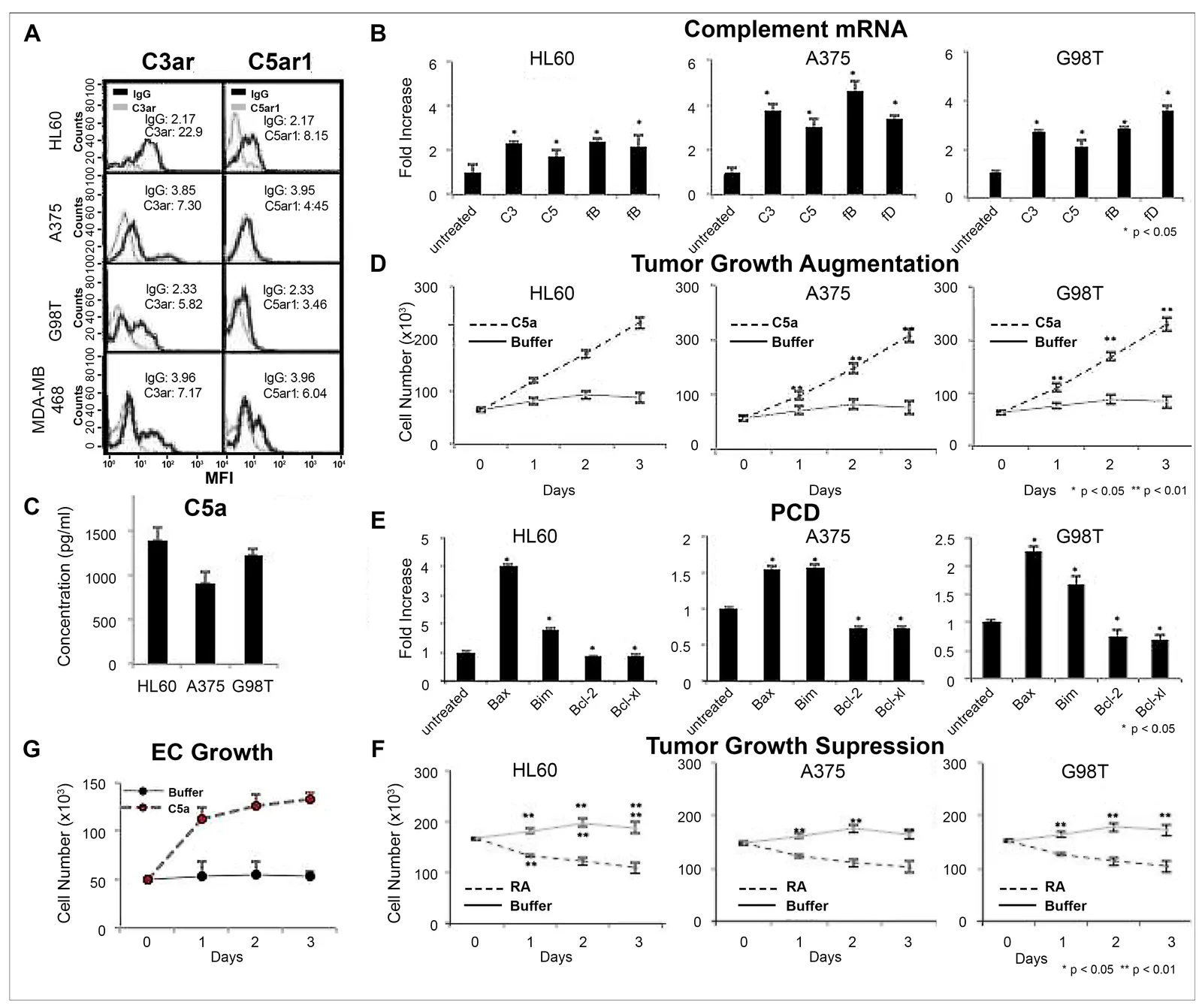
Autocrine C3ar/C5ar1 signaling is growth-inductive in human cancers, and disabling it triggers apoptosis. (**A**) Human HL60 lung cancer, A375 melanoma, G98T glioblastoma, and MDA-MB 468 breast cancer cells were assayed for C5ar1 expression by FACS. MCF is given, IgG is a nonrelevant Ab control. (**B**) The unstimulated human cancer cell lines were assayed for AP complement component mRNA expression by qPCR. (**C**) Their supernatants were assayed for C5a production by ELISA. (**D**) C5a (100 ng/mL) was added to the cell lines equilibrated in 0.5% FBS and cell numbers were quantitated over 3 days. (**E**) The cell lines were incubated for 3 days with or without C3ar-A/C5ar1-A (10 ng/mL each), and their RNA was assayed for anti-apoptotic factors, Bcl-2 and Bcl-xL, and pro-apoptotic factors, Bax and Bim, by qPCR. (**F**) The cell lines were incubated with C3ar-A/C5ar1-A, as in (**E**), and cell numbers were quantitated over 3 days. (**G**) C5a (100 ng/mL) was added to HUVEC equilibrated in 0.5% FCS and cell numbers quantitated over 3 days. * *p* < 0.05, ** *p* < 0.01.

To test whether the results with murine cancers similarly apply to human cancers, we performed the same studies in four human cancers of diverse lineages (HL60 myelomonocytic cells, A375 melanoma, G98T brain cancer, and MDA-MB 468 breast cancer), including two that are not represented in the murine cancers. All human lines similarly expressed C3ar/C5ar1 (Figure 3A). All likewise produced AP (C3/fB/fD) and C5 mRNA transcripts (Figure 3B), endogenously generated C3a [18,19] /C5a (Figure 3C) and grew in response to C5a (Figure 3D). The addition of C3ar-A/C5ar1-A to the culture caused upregulation of Fas/FasL and reciprocal downregulation of Bcl-2/Bcl-xl mRNAs in all (Figure 3E). In line with this, all were significantly growth-inhibited by C3ar-A/C5ar1-A (Figure 3F). The findings for human cancers thus paralleled those for murine cancers.

Tumor expansion depends on tumor-associated angiogenesis. To enable this local angiogenesis, some cancers secrete vascular endothelial cell growth factor-A (VEGF-A) [20,21,22]. In line with our previous findings that VEGF-A’s induction of vascular endothelial cell (EC) growth is interconnected with C3ar/C5ar1 signaling [19], the addition of C5a to non-confluent cultures of human umbilical vein endothelial cells (HUVECs) augmented HUVEC growth (Figure 3G). The growth effect of C5a is consistent with our previous studies of immune cells [7,11] and cancer cell lines [18], as well as ECs in cardiovascular cell settings [23]. Conversely, the addition of C3ar-A/C5ar1-A suppressed the growth of the proliferating HUVEC (not shown) implicating the same signaling pathway in the tumor and its vasculature in promoting tumor growth and the connecting blockade of C3ar/C5ar signaling with concurrently inhibiting both.

### 3.4. Disabling C3ar/C5ar1 Signaling Reduces Tumor Progression in Vivo

**Figure 4:**
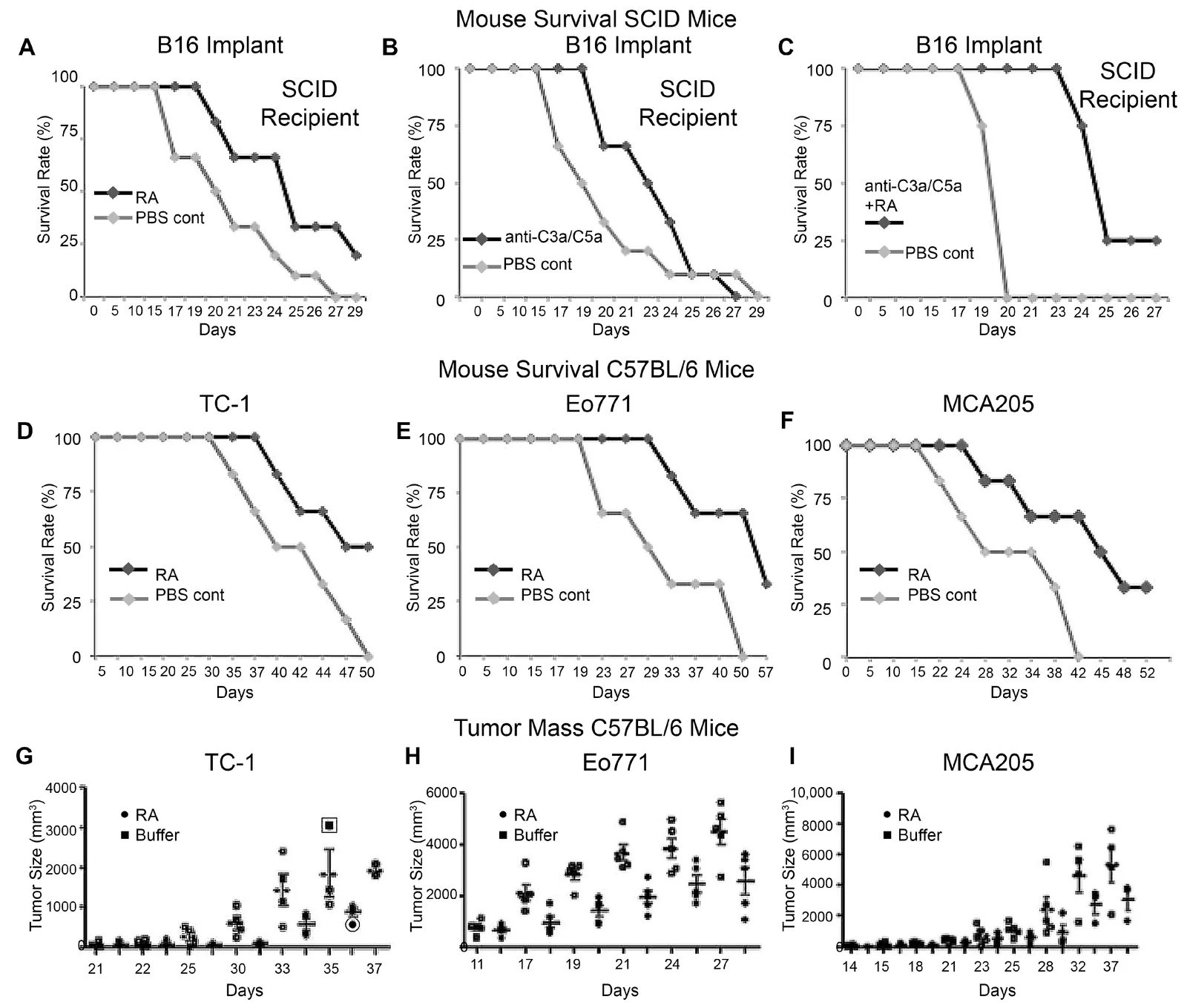
Effect of blockade of C3ar/C5ar1 signaling in cancer cells on tumor progression in vivo. (**A**) B16 melanoma cells (1 × 10^4^ cells) were inoculated s.c. into two matched sets of SCID mice (*n* = 5 per group). One set was treated with C3ar-A/C5ar1-A (RA) (1 mg/kg each) i.p. every other day, and the other was treated with the same amount of MeOH vehicle control. Survival was assessed. The experiment was repeated three times (total *n* = 14 mice, * *p* = 0.0433). (**B**) The protocol used in (**A**) was repeated (*n* = 5 per group), this time administering anti-C5a/anti-C3a mAbs (1 mg/kg each) i.p. weekly. (**C**) The same protocol as in (**A**) was again used, this time administering both C5ar1-A/C5ar1-A and the anti-C3a/anti-C5a mAbs together (*n* = 5 per group). (**D**–**F**) The same protocol as in (**A**) was repeated with TC-1 cells, Eo771 cells, MCA205 cells, and B16 cells (Figure S1B) in WT C57BL/6 recipients using C3ar-A/C5ar1-A and vehicle control. (**G**–**I**) The protocol in (**D**–**F**) with TC-1 cells, Eo771 cells, MCA205 cells, and B16 cells (Figure S1B lower) was followed, this time quantifying tumor size as a function of time.

We next tested whether the above in vitro findings apply in vivo. To eliminate effects of adaptive immune responses, we performed initial studies with SCID mice. We inoculated 10^4^ B16 melanomas s.c. in the SCID mice and administered (1) C3ar-A/C5ar1-A or vehicle control i.p. every other day, (2) anti-C5a/anti-C3a mAbs or isotype controls i.p. weekly, or (3) the antagonists and the mAbs in combination, in conjunction with vehicle-only controls. The receptor antagonists (Figure 4A), as well as the mAbs (Figure 4B), prolonged mouse survival, and the combination (Figure 4C) had the greatest effect. A second set of studies in (isologous) B16-bearing WT (C57BL/6) mice treated with C3ar-A/C5ar1-A showed similarly increased mouse survival (Figure S1B). Similar prolongation of survival in C57BL/6 WT recipients was observed with isologous TC-1 lung carcinoma cells (Figure 4D), Eo771 breast cancer cells (Figure 4E), and MCA fibrosarcoma cells (Figure 4F). Consistent with the increased mouse survival, the in vivo C3ar-A/C5ar1-A treatment decreased TC-1 tumor size by ~60% (Figure 4G), breast tumor size by ~50% (Figure 4H), fibrosarcoma tumor size by ~65%, (Figure 4I), and melanoma size by ~30% (Figure S1B). The inhibition of TC-1 growth by C3ar-A/C5ar1-A occurred in *CD11b^-/-^* mice (deficient in CD11b/CD18-expressing mononuclear cells and polymorphonuclear cells (PMN)), indicative of direct effects on the tumor rather than only on the decreasing recruitment of immunosuppressive MDSCs (Figure S1C). Moreover, tumor growth in *C3^-/-^* recipients was not significantly faster than that in WT recipients (Figure S1), indicative of the C3a/C5a-activating ligands deriving from the cells and not solely from the plasma, which is unable in the case of *C3^-/-^* mice to assemble plasma C3 (C3bBb) and C5 (C3bBb3b) convertases.

### 3.5. Intratumoral Administration of C3ar/C5ar1 Pharmaceutical Antagonists in Poly (Lactic-Co-Glycolic Acid) (PLGA) Causes Robust Tumor Suppression

**Figure 5:**
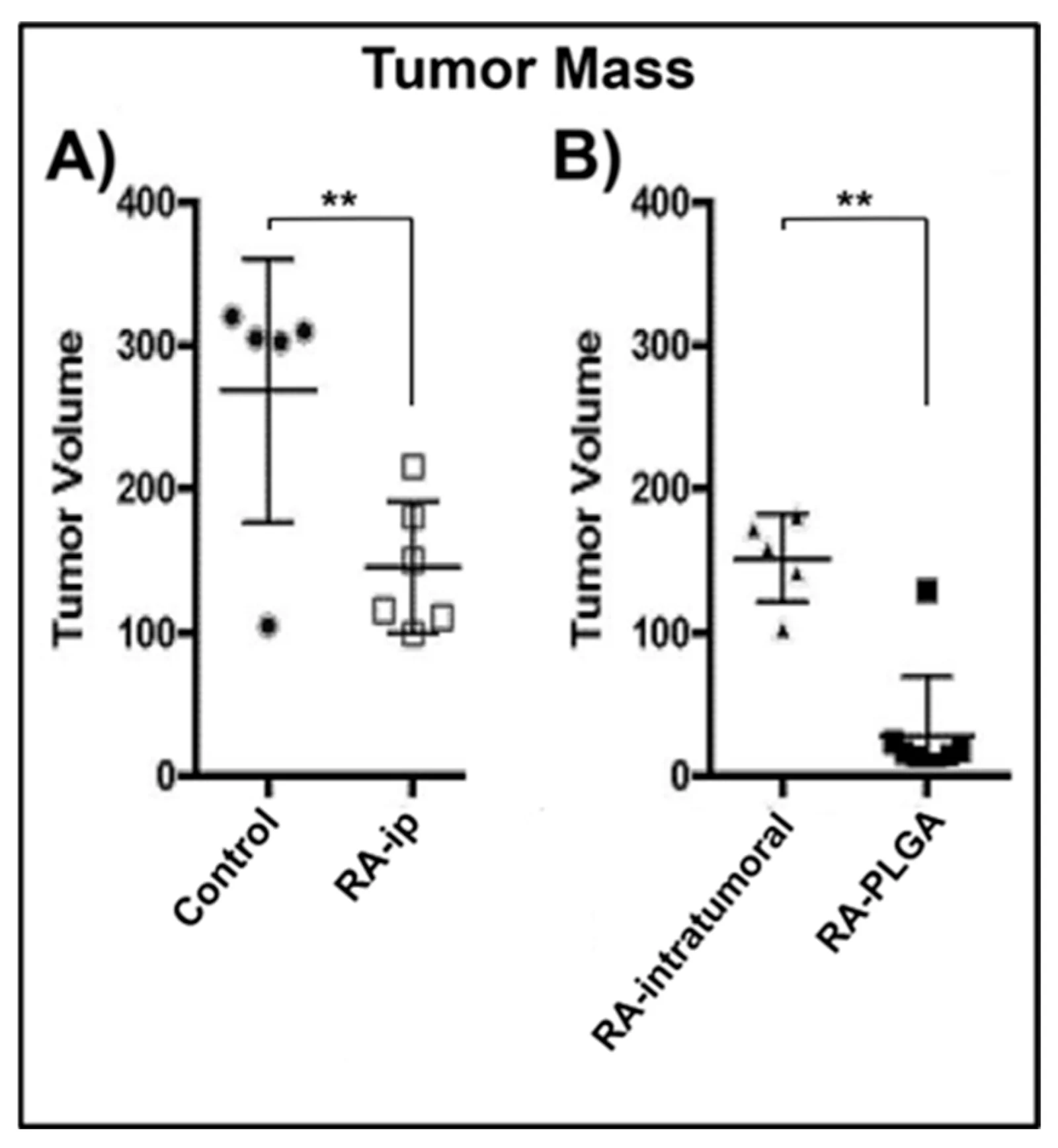
Intratumoral injection of C3ar-A/C5ar1-A in PLGA polymer robustly suppresses cancer growth. (**A**) A total of 20,000 TC-1 cells were implanted s.c. into the backs of C57BL/6 WT mice. Mice (five in each group) were alternatively treated weekly for 6 weeks i.p. with 1) methanol vehicle control (black dot) or 2) C3ar-A/C5ar1-A (1 mg/kg each) (open square) injected i.p. (**B**) In a companion experiment, WT C57BL/6 mice (five in each group) were identically inoculated with the same number of TC-1 cells. The mice (five in each group) were treated weekly with C3ar-A/C5ar1-A (1 mg/kg each) in media (triangle) or in slow-release PLGA polymer (closed square) injected intratumorally. Tumors were harvested at 7 weeks and the tumor mass was measure. ** *p* < 0.025.

Because the above effect of systemic blockade of C3ar/C5ar1 signaling was partial and systemic treatment can affect immune cells and multiple stromal cell types outside of the TME [12,24,25], as well as inhibit the tumor, we hypothesized that focusing and sustaining C3ar/C5ar1 antagonism in the tumor bed would be a preferable to employing a generalized intermittent C3ar/C5ar1 blockade. To examine this, we used C57BL/6 mice as recipients and inoculated them s.c. on the back with 10^4^ TC-1 cells [26,27]. As a baseline enabling comparison to the systemic blockade employed in Figure 4, we first treated groups of the mice (5 each) i.p. with either media alone or C3ar-A/C5ar1-A. We applied the treatments weekly for 6 wks. We examined the mice at 7 weeks for tumor size. Consistent with the above ~50% anti-tumorigenic effect of the C3ar/C5ar1 blockade (Figure 4), the i.p. C3ar-A/C5ar1-A treatments caused ~50% inhibition of the tumor mass (Figure 5A).

In the companion study restricting the C3ar/C5ar1 blockade to the cancer, we injected the tumor-bearing mice intratumorally with 100 ng/mL each of C3ar-A/C5ar1-A in media or in PLGA polymer to focus the C3ar-A/C5ar1-A in the tumor and its vasculature and minimize effects on other cells. We then repeated the protocol in Figure 5A, in which we treated the mice weekly for 6 weeks and measured tumor size at 7 weeks. While the intratumoral treatment with C3ar-A/C5ar1-A in media caused a similar 50% inhibition, the intratumoral PLGA-C3ar-A/C5ar1-A treatment led to near-complete tumor ablation (Figure 5B).

## 4. Discussion

The main finding of this study is that jointly inhibiting autocrine C3ar/C5ar1 signaling in cancer cells and the TME by administering pharmaceutical antagonists intratumorally in an ISFI robustly promotes cancer elimination. It does so by multiple mechanisms. In the cancer, disabling C3ar/C5ar1 signaling is both antimitotic and proapoptotic. In tumor-associated ECs, the disabled C3ar/C5ar1 signaling prevents VEGF-A production [18] and consequent VEGFR2-induced growth signaling [19]. In MDSCs, it circumvents their C3ar1/C5ar1-dependent recruitment [14]. This targeted approach differs from the global inhibition of C5ar1 signaling employed by others [4,8]. Since our studies show that autocrine C3ar/C5ar1 signaling operates in multiple cancer types, this treatment approach, in principle, could have broad translational relevance for solid cancers. While its inhibitory effect on MDSC recruitment and consequent lifting of MDSC immunosuppression confers many elements of “checkpoint therapy”, it provides the potentially added benefits of conferring anti-tumor effects, e.g., induction of cancer cell apoptosis and inhibition of VEGF-A-induced EC growth, that could be beneficial in checkpoint therapy resistant patients.

The first insight deriving from the findings in this paper is that autocrine C3ar/C5ar1 signaling operates broadly in multiple cancer types, and in all cases plays an essential role in their viability, in addition to their proliferation. The C3a and C5a are endogenously produced in the cancer cells, and their intracellular levels, while not precisely known, are amplified with activation [19]. Our analyses of four human and four murine cancers of diverse origins showed that this GPCR signaling uniformly promoted their growth, and that disrupting it consistently triggered their apoptosis by both the *extrinsic* and *intrinsic* PCD pathways. Although our Annexin V data does not distinguish between early and late apoptosis, in vitro and in vivo survival data of the of C3ar/C5ar1-antagonized cells show that they progressively die, and our studies have shown that the disabled C3ar/C5ar1 signaling in the cancer cells lowers the LD_50_ of cytotoxic agents > 10^2^–10^4^ fold (*Pohlmann E and Medof ME, unpublished*). These data directly parallel prior findings implicating autocrine C3ar/C5ar1 signaling in the viability of conventional immune cells both constitutively and during activation [7,11]. While it remains to be determined precisely how this GPCR signaling integrates with other anti-apoptotic signaling pathways in cancers, the data indicate that blockade of C3ar/C5ar1 signaling can be effective, irrespective of varying molecular alterations in the different cancer types studied. Previous studies in immune cells mechanistically tied autocrine C3ar/C5ar1 transduction to the activation of PI-3Kɣ, which increases the assembly of PIP_3_ needed for phosphorylation of AKT and its downstream signaling to mTOR [7,12]. Measurements of p-AKT in murine B16 melanomas and MCA205 sarcomas in this study documented the same C3ar/C5ar1 signaling requirement for AKT activation in these cancer cell types. Our prior immune cell studies [28] provided data that co-ligation of the C3ar/C5ar1 GPCRs also promotes activation of NF-kB, ERK, STAT3 and PLC, all of which are widely connected with cancer cell proliferation. While it is well established that the PI-3K-AKT-mTOR pathway is central to the growth of many cancers, sustained PIP_3_ assembly has been widely attributed solely to activated PI-3Kα which generally is connected with receptor tyrosine kinase (RTK) signaling. The studies herein argue that joint PI-3Kɣ and PI-3Kα activation is required to sustain this viable, growth-inductive pathway. A corollary finding in the studies herein is that C3ar/C5ar1 signaling operates tonically in the absence of serum factors to prevent apoptosis in the cancer types studied, as found in immune cells.

The second insight derived from the findings in this paper is the C3a/C5a fragments that have been implicated in affecting cancer outcomes derive from C3/C5 proteins produced by the tumor and cells in the TME rather than solely from (liver-produced) C3/C5 proteins in plasma. Although the inhibitory effect of anti-C3a/C5a mAbs on tumor growth in vivo could reflect the inhibition of C3a/C5a generated from plasma C3/C5 protein, it also could reflect the inhibition of cellularly derived C3a/C5a ligated to C3ar/C5ar1 on the cell surface. In support of the latter, tumor growth in WT and *C3^-/-^* mice did not significantly differ. Further studies will be needed to definitively clarify this issue.

Tailoring the delivery of the C3ar/C5ar1 antagonists for targeted release in the tumor by administering them in an ISFI [29] markedly strengthened tumor inhibition as compared to systemic i.p. or direct intratumoral C3ar-A/C5ar1-A administration. We cannot fully explain why the ISFI administration was markedly more effective than the direct intratumoral C3ar-A/C5ar1-A administration in media. Possibilities include locally higher concentrations of the C3ar-A/C5ar1-A; a sustained and uninterrupted, rather than periodic, blockade; avoidance of effects on other cell types; or a difference in the way the antagonists are presented to the tumor and/or its associated cells in the TME. Studies by others [30] have shown that intratumoral administration of controlled-release drug formulations in ISFIs [29] can sustain locally elevated drug levels in the tumor with limited systemic involvement [31,32,33,34]. ISFIs consist of a solution or suspension of matrix (e.g., biodegradable polymer or hydrogel) and active agent(s). Once injected, the liquid solidifies into an implant in response to a stimulus, such as water influx, via the process of precipitation, cross-linking, or polymerization. In this paper, we formulated an ISFI by adding PLGA polymer [29,35] to the solvent 1,2-N-methyl pyrrolidone (NMP) creating a low-viscosity polymer solution that we then mixed with C3ar-A/C5ar1-A. The intratumoral delivery of the C3ar-A/C5ar1-A in PLGA maximized the inhibitory effects of the antagonists on the tumor and minimized their effects systemically. The locally sustained intratumoral delivery also provides continuous suppression of local tumor-associated angiogenesis needed for tumor growth [20,21,22]. This fits with our prior findings [19] that the C3ar/C5ar1 blockade markedly suppresses the augmentation of EC growth by VEGF-A, which several studies have shown is endogenously generated by the tumor to support its angiogenesis. Our previous studies [19] showed that C3ar/C5ar1 proteins are physically associated with VEGFR2 and IL-6 receptor (IL-6R)-gp130 in a signalosome and that joint signaling of all partners is required for VEGF-A production and EC growth. This VEGF-A-inhibitory effect, in principle, could be particularly relevant for solid cancers because drug or immune cell access to cells in the tumor interior is restricted, whereas O_2_ and nutrient deprivation can kill them. Further studies will be needed to test this. In line with this, while chimeric antigen receptor (CAR) T-cell therapy is highly effective against hematological malignancies in which the cells grow diffusely, it is less effective against malignancies that expand as solid tumors.

The third insight coming from the findings in this paper is the C3ar/C5ar1 blockade strategy can circumvent the accumulation of tumor-associated MDSCs in the TME by virtue of inhibiting their recruitment. A large body of data previously implicated tumor-induced Tregs in tumor escape from immune attack. Checkpoint therapy against proteins connected with Treg induction or function, e.g., PD-L1 or CTLA-4, has yielded dramatic anti-tumor effects in multiple tumor types [36,37]. A downside, however, has been that the global blockade of these checkpoint proteins induces autoimmune complications [38,39]. The ability of the C3ar/C5ar1 blockade in PLGA to eliminate MDSC recruitment is in line with our past findings that the MDSC influx needed for transplant tolerance and survival is dependent on MDSC C5ar1 signaling [14]. These data add to the abundant evidence that the tumor-associated MDSCs inhibit anti-tumor Teff responses. Our prior studies [12,13,14,40] showed that the C3ar/C5ar1 blockade prevents the generation of IL-23 connected with the augmentation of cancer cell growth, as well as IL-1β, IL-6/IL-8 and STAT3 connected with MDSC recruitment and function. In principle, it also could inhibit fibroblast growth in the TME [18,41].

This combined anti-MDSC tumor recruitment, together with the proapoptotic effect of the C3ar/C5ar1 blockade in cancer cells, raises the possibility of significant tumor suppression in cancer types that present as solid tumor masses [19]. The unique property of the ISFI containing C3ar/C5ar1 pharmaceutical antagonists employed in this study is that the tumor, its vasculature, and MDSCs can be jointly and simultaneously inhibited. Its use differs from that of an ISFI containing a cytotoxic drug in that it directly targets multiple processes in different cell types that physiologically promote tumor growth. It is noteworthy that the growth linkage of C3ar/C5ar1 signaling in cancers could be relevant to the unanticipated tumor promoter effects of treatments employing mAbs directed against cancer cell surface proteins. If the administered mAb is an activator of a systemic complement, it could generate C3a/C5a from plasma C3/C5 which, in principle, could promote C3ar/C5ar1 signaling in the cancer cells, as well as recruit MDSCs, thereby favoring tumor cell viability and proliferation. It could also promote tumor-associated angiogenesis, which could further add to the pro-tumor effect.

Studies over several years by several groups provided data pointing to C5ar1 (and/or C3ar) signaling being involved in tumor growth. They did not, however, tie it to cancer cell viability and tumor-associated angiogenesis or connect its inhibition with interference in MDSC recruitment. Both tumor intrinsic and tumor extrinsic effects were documented. Among the intrinsic effects, both early and more recent studies [42,43] detected C5ar1 expression on various cancer cell types. Consistent with its signal transduction being cancer growth relevant, adding C5a to in vitro cultured cancer cells was found to induce the synthesis of multiple proteases including matrix metalloproteinases (MMPs) [26,44], evoke increased cancer cell motility in Matrigel [43], and promote cancer cell chemotaxis [45,46,47,48]. One study found that C5a augmented the proliferation of human nasopharyngeal carcinoma cells and activated STAT3 [48], but assumed that the C5a was derived solely from plasma C5. Among the *extrinsic* effects is the connection of C5a with cancer cell recruitment of MDSCs that inhibit anti-tumor CD8^+^ cell cytotoxicity [26]. The source of the C5a, however, remained controversial. One study provided evidence that the cancer cells generate C5a from plasma C5 via the elaboration of a membrane protease [47]. C5a release from a C5a-transfected cancer was found to induce VEGF-A capable of augmenting tumor-associated angiogenesis, but how the VEGF-A was produced was not clarified [49]. Positive vs. negative effects on tumor growth of high vs. low C5a production by a tumor transfectant were reported [49]. In line with C5a having autocrine growth effects in cancers [50], it was reported that C5a augmented AKT phosphorylation in breast cancer cells and enhanced their growth [46], but the study presumed that the C5a derived from plasma C5 [46]. The literature thus pointed to C5a having both positive and negative effects on tumor progression, and the source of the C5a was not clarified.

The connection of C5a with recruitment of MDSCs to the tumor has been highlighted in recent studies by multiple groups [26,51,52,53]. In some of these studies, C5a’s effect has been attributed solely to this process [54,55,56]. The effects of autocrine C5ar1 signaling in the tumor and/or its vasculature have been incompletely characterized. While the studies herein are in line with the important pro-tumor effect of tumor-associated MDSCs, our findings that many cancer types endogenously produce C3a/C5a and that autocrine C3ar/C5ar1 signaling, in both cancer cells and ECs, function to promote tumor viability and growth, supporting a multilateral rather than a unilateral protumor effect of C5a.

The C3ar/C5ar1 signaling in MDSCs, enabling their tumor influx, seemingly differs from that in conventional monocytes/macrophages described in previous studies. Our previous studies showed that potentiated C3ar/C5ar1 signaling in CD11b^+^ F480^+^ macrophages or in CD11c^+^ DCs downregulates immunoinhibitory PD-L1 and ICOS-L expression, and reciprocally upregulates B7/CD40 costimulatory molecule expression [13]. Although we are not yet able to account for the difference, one possibility is that C3ar/C5ar1 signaling is needed for the mobility of undifferentiated mononuclear cells/macrophages, and their polarization is imprinted by the target cells that attract them. A second possibility is that monocytes/macrophages are tonically immunosuppressive by default and require a proinflammatory signal to mature them. Both possibilities would be in line with the known plasticity of these cell types. It is also in line with the fact that TLR signaling biases between tolerogenic and proinflammatory phenotypic properties, and the finding that undifferentiated MDSCs jointly express proteins with phenotypically opposing functions, e.g., both (M1-type) iNOS and (M2-type) arginase [57].

The potent tumor-suppressive effects of the anti-cancer strategy described herein raise the question of how it can be translated clinically in patients. Because the above experiments showed that tumor viability and growth, its associated angiogenesis, and immunosuppressive MDSC recruitment all depend on C3ar/C5ar1 signaling, and as this dependence is a common feature of many cancer cell types, the use of C3ar/C5ar1 antagonists in an ISFI could be effective when there is a solid tumor mass that can be targeted. An approach for disseminated tumors could be to administer C3ar-A/C5ar1-A (or C3ar/C5ar1 siRNAs) in lipid nanoparticles targeting a tumor antigen such as PSMA in prostate cancer. In support of the potentially wide applicability of this treatment approach, our experiments (*Medof ME, unpublished* [58]) have found that C3ar/C5ar1 antagonism not only inhibits the viability and growth of the eight cancer lines studied herein, but similarly affects six additional cancers of diverse origins, i.e., all lines studied. Because our prior studies [18] have shown that the blockade of this GPCR signaling confers dominant suppression on PIP_3_ assembly, as well as AKT and STAT3 signaling, leveraging it in the case of solid tumors with or without a tumor-appropriate cytotoxic agent included in the ISFI could improve the chances for a successful treatment outcome.

## Data Availability

Available through contacting the corresponding author.

## Supplementary Materials

The following supporting information can be downloaded at https://www.mdpi.com/article/10.3390/cells15110971/s1, Figure S1: TC-1 cells incubated with and without C3ar-A/C5ar1-A (10 ng/mL each) were assayed for expression of Fas and Fas-L by FACS.
