# Supplementary material for: Intratumoral C3ar/C5ar1 Antagonists Imbedded in an In Situ Forming Implant Can Robustly Suppress Solid Tumors
Source: Cells. 2026 May 25;15(11):971. doi: 10.3390/cells15110971 (PMC13256460; doi:10.3390/cells15110971)
Supplement: Supplementary file 1 [file cells-15-00971-s001.zip › cells-4055677-supplementary.pdf]

SUPPLEMENTARY FIGURE

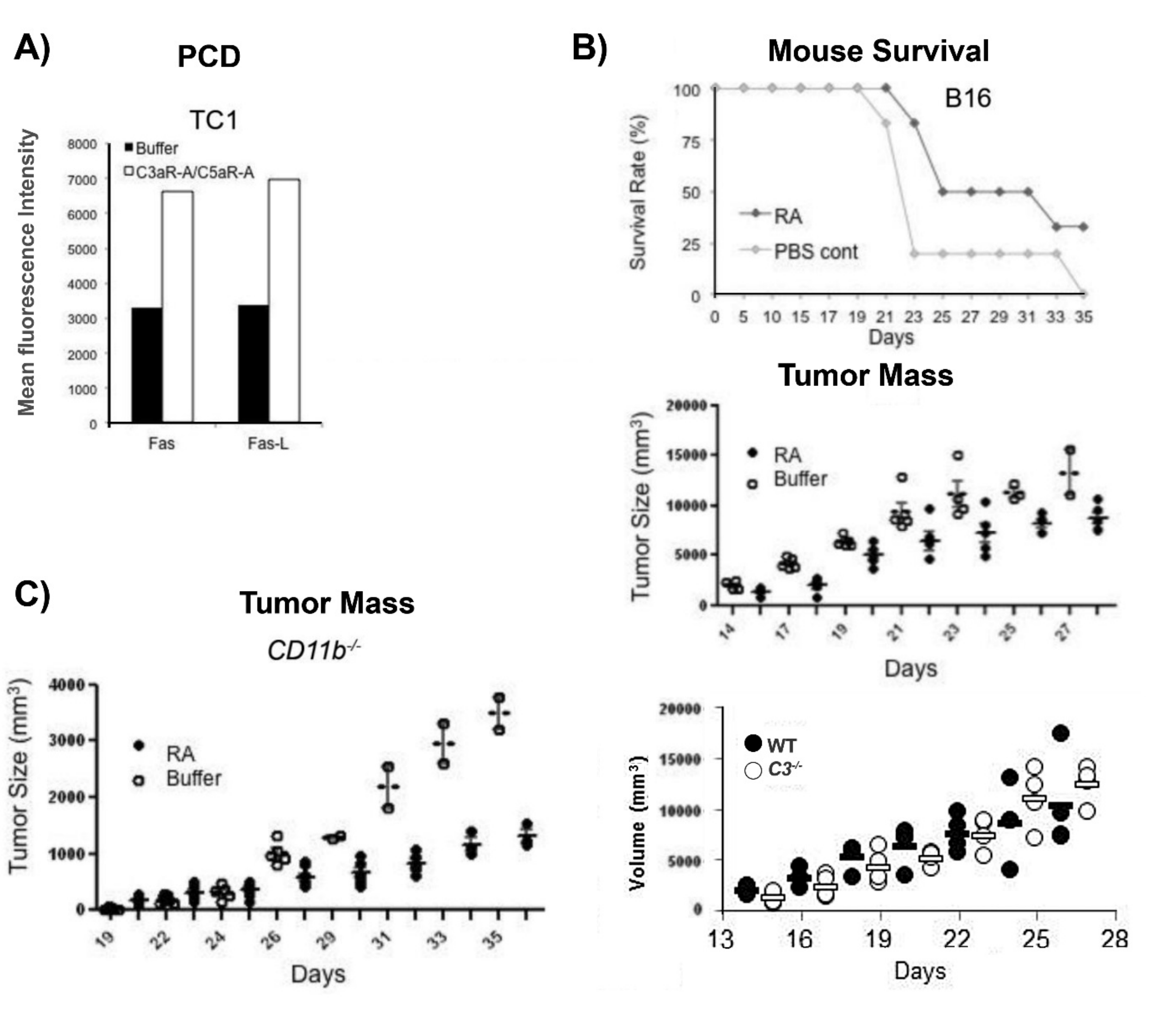

Figure S1

Figure S1. A: TC-1 cells incubated with and without C3ar-A/C5ar1-A (10 ng/ml each) were assayed for expression of Fas and Fas-L by FACS. Bars represent mean fluorescence intensity (MFI) data from FACS analysis. **B**. (upper) WT C57BL/6 mice were inoculated with 1x10^4 cells (B16; n=5 per group) and treated i.p. with C3ar-A/C5ar1-A (RA) (1 mg/kg) or PBS (MeOH) and survival was assessed**.** (lower) Tumor size in C57BL/6 WT mice inoculated with B16 cells (n=5 per group) and treated as above was quantified as a function of time. **C**. (left) *CD11b*$^{-/-}$ mice inoculated with 1x10^4 cells (TC-1; n=6 per group) were treated i.p. with C3ar-A/C5ar1-A (RA) (1 mg/kg) or PBS (MeOH) and tumor size was quantified as a function of time. (right) WT or *C3*$^{-/-}$ mice (4 each group) were inoculated s.c. with 1x10^4 B16 cells and tumor growth was measured daily.
